# Understanding Climate Change Threats to Vertebrate Wildlife by Studying Ecoimmunology Across Biological Scales

**DOI:** 10.1093/icb/icaf150

**Published:** 2025-08-20

**Authors:** Anna C Fagre, Daniel J Becker, Laura A Pulscher, Molly C Simonis, Colleen G Duncan

**Affiliations:** Department of Microbiology, Immunology, and Pathology, Colorado State University, Fort Collins, CO, 80523USA; Department of Veterinary Microbiology and Preventive Medicine, Iowa State University, Ames, IA, 50011USA; School of Biological Sciences, University of Oklahoma, Norman, OK, 73019USA; Department of Microbiology, Immunology, and Pathology, Colorado State University, Fort Collins, CO, 80523USA; College of Forestry, Wildlife and Environment, and Department of Pathobiology, Auburn University, Auburn, AL, 36849USA; Department of Microbiology, Immunology, and Pathology, Colorado State University, Fort Collins, CO, 80523USA

## Abstract

Climate change threatens organismal health and ecological stability in myriad ways, the impacts of which are often difficult to characterize given their complex and interacting nature. To facilitate comparisons across taxa and ecosystems, we discuss the importance of a cross-scale approach to better characterize the ways in which climate change processes threaten wildlife immunity. Centering available examples from the vertebrate wildlife literature, we supplement with examples from the livestock literature to illustrate ways in which abiotic stress impacts immunity from molecular to community scales of biological organization. To highlight opportunities for cross-scale integration, we present a series of vignettes—drought, temperature extremes, storms and flooding, and habitat alterations and shifts—prior to discussing the complexities inherent to studying multiple interacting threats using heavy metal contamination as an example. Finally, we outline mechanisms by which collaborations across disciplines and sectors can continue strengthening capacity for studying the drivers of climate change-associated threats to wildlife immunology.

## Introduction

Climate change presents a multifaceted threat to ecosystem stability and organismal health. Increased temperatures, altered precipitation, and shifting weather patterns—collectively referred to herein as abiotic stress—can disrupt physiological homeostasis and drive ecological perturbations (Watts et al. 2015). These stressors alter habitat use and phenology, disrupt life history timing, and reshape interspecies interactions; often resulting in immunosuppression and enhanced pathogen transmission (Becker et al. 2019; Schatz and Park 2021; Zhang et al. 2022; Eby et al. 2023). While prior reviews describing climate change impacts on wildlife have focused on disease susceptibility, pathogen transmission (Becvarik et al. 2023; Plaza et al. 2024), system-specific outcomes (e.g., cognition (Soravia et al. 2021), or reproduction (Hansen 2009; Andreasson et al. 2020)), relatively few have focused on the immunological consequences of abiotic stress.

Efforts to define and assess wildlife health have historically lagged parallel work in human health and veterinary medicine, in which formalized frameworks are more established (Acevedo-Whitehouse and Duffus 2009; Stephen 2014; Wittrock et al. 2019; Greening et al. 2025). As wildlife health practitioners across disciplines work to better integrate complex interactions between wildlife and their environments, ecoimmunology has emerged as a valuable lens through which to evaluate such parameters. This interdisciplinary framework examines the coordination among immune, nervous, and endocrine systems in coordinating responses to environmental and physiological stressors, leveraging methods from across disciplines to characterize how such stressors shape infection risk (Graham et al. 2011; Schoenle et al. 2018; Martin et al. 2021; Ohmer et al. 2021; Assis et al. 2022). Notably, species-specific traits like metabolic strategy, thermal tolerance, and life history can influence vulnerability to immunological homeostasis. This further complicates efforts to compare impacts across systems (Zuk and Stoehr 2002; Viney et al. 2005).

We address this gap by integrating elements of the International Union for the Conservation of Nature (IUCN)—Conservation Measures Partnership (CMP) unified classification of direct threats (v3.3) to frame climate-driven exposures in vertebrate wildlife species (Salafsky et al. 2008), highlighting mechanisms by which these exposures interact with ecological hazards to threaten wildlife immunity. We begin by providing a brief overview of vertebrate immune function; emphasizing how stress dysregulates both innate and adaptive immune responses.

## The vertebrate immune system

The vertebrate immune system is a complex network of coordinated systemic and local responses that aim to differentiate “self” from “non-self,” expeditiously detecting and eliminating the latter (Boehm 2025). It is orchestrated by immune cells (i.e., leukocytes) and the signaling molecules they use to coordinate their defensive activities. The vertebrate immune system consists of primary and secondary lymphoid organs (which vary by species; summarized in Table 1 of Zimmerman et al. 2010), and it transports leukocytes throughout the body via the lymphatic system. For those with limited backgrounds in immunology, review articles overviewing the vertebrate immune system have been published (Buchmann 2014; Downs and Stewart 2014; Riera Romo et al. 2016; Schoenle et al. 2018; Boehm 2025).

### Innate immunity

The innate immune system serves as the body's first line of defense against potential invaders—it acts quickly (within minutes to hours) and without specificity. Innate immune defenses include anatomical barriers (e.g., skin, mucosal tissue), chemical barriers (e.g., low pH, lysozyme), soluble signaling molecules (e.g., cytokines, chemokines), and cellular defenders (e.g., natural killer cells, neutrophils in mammals (heterophils in birds) (Udino et al. 2024)). External anatomical barriers can be compromised by injury and trauma (e.g., laceration) or through chemical/topical tissue damage (e.g., contact dermatitis, burns), predisposing individuals to secondary infections. Internal anatomical barriers (e.g., mucosal tissue, respiratory epithelium) can similarly be disrupted by exogenous stressors, with their compromise predisposing individuals to opportunistic infections (e.g., “leaky gut” in heat-stressed cattle) (Koch et al. 2019; Cantet et al. 2021; Mettelman et al. 2022).

### Adaptive immunity

The adaptive (or acquired) immune system is pathogen-specific and leverages immune memory to neutralize invaders. It can be further divided into cell-mediated immunity (T cells) and humoral immunity (B cells) (Sebina and Pepper 2018; Garcia 2019). Cell-mediated immunity (CMI) is comprised of three different kinds of T cells: cytotoxic lymphocytes (T_C_; also referred to as killer T cells or CD8^+^ T cells), T helper cells (T_h_; also referred to as CD4^+^ T cells), and regulatory T cells (T_reg_). T_h_ cells help activate their cytotoxic counterparts by secreting cytokines after they encounter an antigen-presenting cell (APC). After the APC engulfs a pathogen, peptides derived from the antigens are presented on the cell surface by the major histocompatibility complex (MHC) (Cortazar‐Chinarro et al. 2025). The second component of the adaptive immune system, humoral immunity, is driven by B cells and antibodies they produce in response to antigen encounters. Aided by T cells, B cells differentiate to prioritize secretion of antigen-specific antibodies for pathogen neutralization and clearance.

### Stress and the immune response

Stress activates the hypothalamic–pituitary–adrenal (HPA) axis, stimulating the release of glucocorticoids (GCs). Over time, the protective feedback of GCs weaken, and prolonged GC elevation leads to systemic inflammation and immunosuppression (with impacts to both innate and adaptive responses varying across species and by acuity) (Park et al. 2021; Zhou et al. 2021; Koch et al. 2023). Relevant to ecoimmunology studies is the concept of T_h_ cell polarization, in which signaling molecules (e.g., GCs) shape the relative proportions of T_h_1 cells (promoting cell-mediated immunity) and T_h_2 cells (promoting humoral immunity) (Demas and Nelson 2012). While acute stress can stimulate the immune response, prolonged release of GCs drives inflammation and dysregulation of leukocyte responses (e.g., T cell polarization favors T_h_2 > T_h_1), dampening CMI responses (Elenkov and Chrousos 1999; Berger 2000).

## Studying the immune consequences of abiotic stress across scales

Abiotic stressors associated with climate change disrupt immunological homeostasis across biological scales—from altering the expression of non-coding RNAs to driving shifts in pathogen-sharing networks (Van Woesik et al. 2022). A recent analysis seeking to broadly identify themes in climate change studies pertaining to wildlife health describes a preponderance of papers focusing on temperature and its effects (Greening et al. 2025). Commonly cited among these are increases in temperature and alterations in weather patterns, which together drive increased frequency of extreme weather events, floods, and droughts (Watts et al. 2015). In this section, we illustrate the immune consequences of climate change on vertebrate wildlife across biological scales using four vignettes: drought, temperature extremes, storms and flooding, and habitat alteration and shifts. Selection of these categories was guided by the IUCN–CMP Classification of Direct Threats (v3.3) level 2 threats within the level 1 entry “11. Climate Change” (Salafsky et al. 2008). We acknowledge that in the recently released IUCN-CMP Threats (v4.0) (Salafsky et al. 2025), a separate level 1 category (“10. Natural Disasters”) now captures storms and flooding under “10.2 Extreme weather events.” We also note that while “11.1 Habitat alteration and shifts” is listed first among the v3.0 level 2 threats, we present it at the end of this section to reflect its position as an indirect driver of immune disruption. We emphasize that the factors driving such processes occur through a combination of biophysical stressors and, in the following section, we use heavy metal contamination as a case study to illustrate the cascading risks and context-dependent effects that mediate exposure to other threat pathways.

### Drought

Drought is defined by the Intergovernmental Panel on Climate Change (IPCC) as a period during which rainfall is lower than what is historically expected for a given time and season (Calvin et al. 2023). At the organismal level, dehydration results in low fluid volumes and electrolyte imbalances that, over time, can damage the kidneys and result in multi-organ failure (Albright et al. 2017; Jacobs et al. 2020; Elbashir et al. 2023). These impacts extend to the immune system, differentially impacting species with life histories tightly coupled to their environment. In birds experiencing drought conditions, some studies have identified decreased CMI responses (Fair and Whitaker 2008; Stanek et al. 2022). In amphibians, the skin microbiome plays a critical role in regulating host defenses against fungal pathogens like *Batrachochytrium dendrobatidis* (*Bd*). One study examining microbial communities on the skin of pumpkin toadlets (*Brachycephalus rotenbergae*) in the weeks following drought demonstrated marked changes in their microbiome, which coincided with increased *Bd* loads (Buttimer et al. 2024). It is also thought that amphibians may be particularly vulnerable to *Bd* infection during metamorphosis—a transitional period during which their lymphoid tissue undergoes rearrangement and their immune system is compromised (Kohli et al. 2019).

**Fig. 1:**
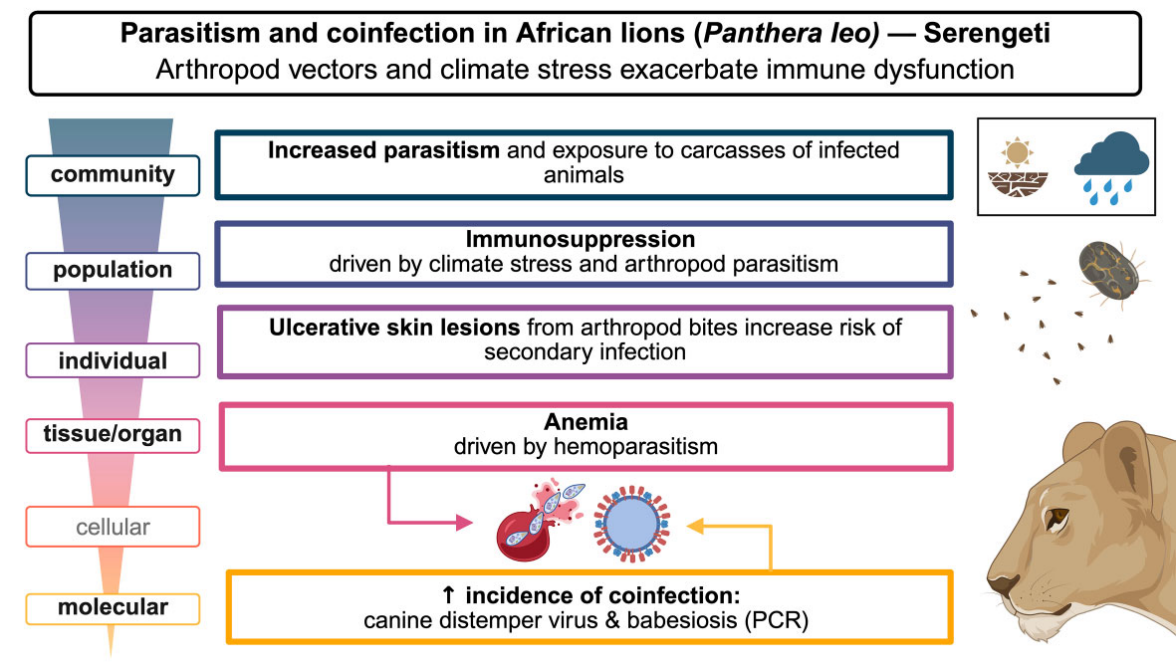
Coinfection and immune compromise in African lions (*Panthera leo*) under climate stress and increased abundance of hematophagous arthropods. Extreme drought conditions followed by heavy rainfall triggered starvation and heavy tick infestation in herbivores, such as Cape buffalo (*Syncerus cafferi*). As African lions preyed on the herbivores, the ixodid ticks likely switched hosts and began feeding on the lions. Underlying immunosuppression, fueled by climatic conditions and ongoing canine distemper virus (CDV) circulation, exacerbated the clinical impacts of tick-acquired babesiosis. Created in BioRender.

Resource shortages caused by drought also change behavior of individual animals and populations, resulting in altered interactions between species and increasing pathogen exposure risk. For instance, a study examining waterhole use patterns in the East African Savanna revealed a higher concentration of parasites during drier periods (Titcomb et al. 2021). Arthropod vector populations (e.g., mosquitoes, ticks) are also influenced by rainfall patterns. Analyses attempting to identify risk factors for severe West Nile virus transmission often identify prior drought years as a significant predictor of emergence in humans (Paull et al. 2017). Potentially exacerbating the risk of heightened pathogen transmission, some studies suggest that drought and post-drought periods correspond with increased abundance of arthropod vector populations (e.g., mosquitoes, ticks). For instance, flea abundance on prairie dogs was higher in drought years than in years with average rainfall, potentiating the risk of plague transmission (Eads et al. 2016). Similarly, Serengeti lions experienced an increased burden of tick infestation after a period of extreme drought followed by heavy rainfall (Fig. 1) (Munson et al. 2008; Nikolin et al. 2017; Weckworth et al. 2020).

Often, drought coincides with periods of abnormally elevated temperature. Both processes impact relative humidity, water availability, and an individual's hydration status. Attempts to thermoregulate using evaporative cooling (e.g., wing fanning, ear flapping) can exacerbate dehydration and potentiate the systemic effects of heat stress on immunity (Cryan and Wolf 2003; Albright et al. 2017; van de Ven et al. 2019).

### Temperature extremes

**Fig. 2:**
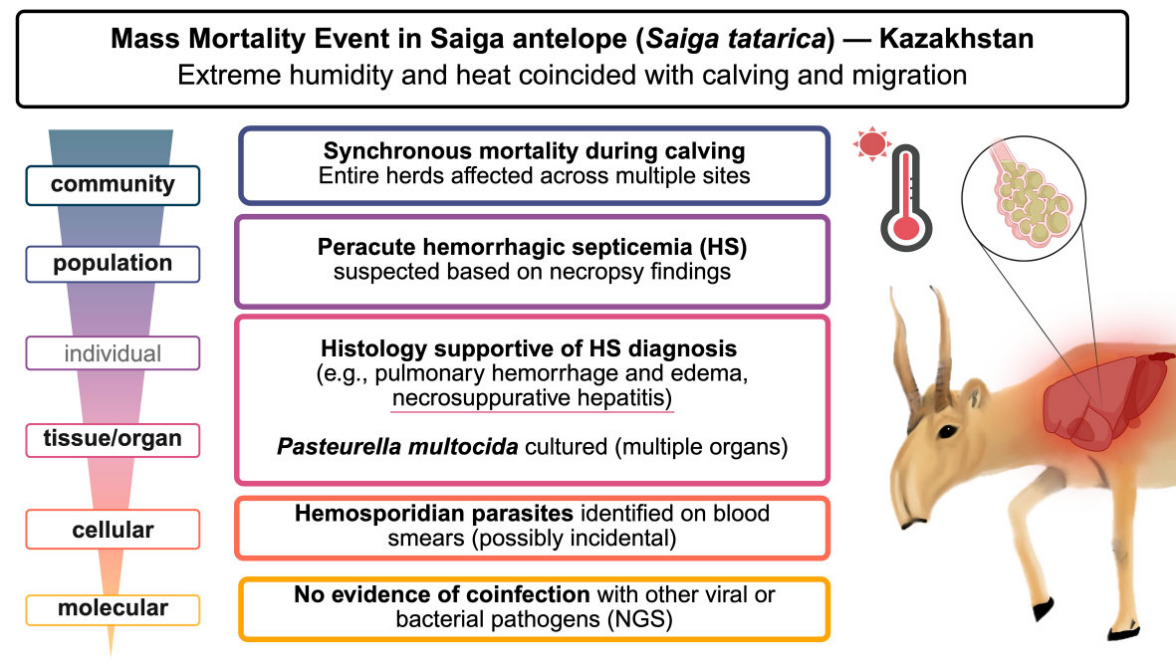
Mass mortality event in Saiga antelope (*Saiga tatarica tatarica*) following extreme weather and calving stress in Kazakhstan. A synchronized die-off occurred during a period of calving and migration immediately preceded by extreme humidity and heat. Clinical signs of systemic illness—characterized by rapid collapse and death of adults followed by starvation and/or later death of neonates—accompanied postmortem findings consistent with hemorrhagic septicemia. *Pasteurella multocida* was cultured from multiple tissues and next-generation sequencing ruled out other major pathogens as coinfecting etiologic agents. Created in BioRender.

In the face of elevated temperature, the severity and chronicity of thermal stress plays an important role in shaping the immune response. Under transient hyperthermia—such as that observed during physiological fever—thermal stress can be immunostimulatory, driving increases in lymphocyte trafficking and antigen presentation. In contrast, prolonged or severe heat stress—the temperature threshold of which will vary by species—dysregulates the immune system via excessive GC release and systemic inflammation (Cantet et al. 2021; Balakin et al. 2025; Wilander and Rathmell 2025). Heat stress has also been shown to compromise important physical barriers. The respiratory epithelium can also be damaged by increased temperature (and/or decreased humidity), potentiating the risk of pneumonia and other respiratory infections. Increased risk of bacterial pneumonia in bighorn sheep (*Ovis canadensis*) and Saiga antelope (*Saiga tartarica*) following extreme heat events and associated ecological shifts (i.e., humidity) are well-documented (Kock et al. 2018; Fereidouni et al. 2019; Orynbayev et al. 2019; Bowen et al. 2022) (Fig. 2).

CMI is also impacted by thermal stress, as T_h_ cell polarization witnesses a shift favoring T_h_2 responses. Compromised T_h_1 responses result in weakened defenses against intracellular pathogens (Yadav et al. 2024; Shi et al. 2025). In free-ranging animals, thermal stress can exacerbate nutritional stress in multiple ways—not only by limiting plant growth but also by shaping foraging behavior and feeding ecology. For example, brushtail possums decreased their protein intake when exposed to different ambient temperatures (Beale et al. 2023). Limitation of energy and micronutrients available to the immune system disrupts homeostasis and compromises both innate and adaptive immune responses (Landete-Castillejos et al. 2002; Rosen and Trites 2005; Deschner et al. 2012; Grueber et al. 2018; Holden et al. 2019).

### Storms and flooding

Storms and flooding can displace wildlife from their native habitat either abruptly (i.e., extreme weather events) or gradually due to habitat loss (i.e., flooding). Storms also contribute to habitat degradation (e.g., nutritional stress, displacement), the consequences of which will be discussed in the following section. The impacts of such occurrences on wildlife health can be difficult to document and require an understanding of pre-disturbance population dynamics. Studies examining wildlife populations in the months following tropical storms suggest that these occurrences are associated with decreased body condition score, decreased population sizes, vagrancy, increased parasitism, and higher pathogen prevalence (Dionne et al. 2008; Cohen et al. 2015; Tonelli et al. 2023; Fisheries 2025).

In addition to the risk of trauma and injury associated with extreme weather events, storms and flooding increase the risk of exposure to water-borne pathogens, irritants, and environmental toxins—including those associated with spoiled silage, many of which are potent immunosuppressants (i.e., aflatoxins) (Dunham et al. 2017; Focker et al. 2023; Casu et al. 2024; Saha Turna et al. 2024). The accumulation of sewage and agricultural runoff, including animal waste, compromise water quality and further exacerbate exposure to water-borne pathogens. Of note, ultraviolet radiation is less effective at inactivating pathogens in surface waters following abnormal precipitation (Williamson et al. 2017). Disease outbreaks associated with flooding events have been linked to several different pathogen groups—bacterial (i.e., *Leptospira* spp., *Vibrio* spp., and members of family *Enterobacteriaceae*—including *Eschirichia coli*), protozoal (*Cryptosporidium* spp., *Giardia* spp.), and viral (i.e., noroviruses, enteroviruses, hepatitis E) (ten Veldhuis et al. 2010; de Man et al. 2014; Natalini et al. 2021).

Standing water also serves as breeding grounds for arthropod vectors of pathogens, such as those that transmit arboviruses (i.e., dengue virus) and protozoa (i.e., haemosporidian parasites) (see, for example, (Coalson et al. 2021)). Increased contact of wildlife with humans, livestock, and other domestic animals is an oft-cited consequence of abrupt environmental degradation, potentiating the risk of cross-species pathogen transmission. For instance, flooding has contributed to the movement of *Toxoplasma gondii* from terrestrial animals to aquatic ecosystems, a well-documented system in which domestic felids are incriminated (VanWormer et al. 2016).

Standing water during post-flood periods is also associated with damage to protective anatomical barriers (e.g., skin, mucosa). Specifically, cases of irritant contact dermatitis have been reported, driven by prolonged exposure to water contaminated with pesticides, sewage, fertilizer, and other waste (Huang et al. 2016). This compromise of the skin barrier further exacerbates the risk of skin and soft tissue infections by bacteria (i.e., *Dermatophilus congolensis* (dermatophilosis/’rainscald’), atypical mycobacteria, *Pseudomonas aeruginosa, Staphylococcus aureus*) and fungi (i.e., aspergillosis, mucormycosis, dermatophytosis). A review examining reported causes of dermatitis in captive and free-ranging wildlife described *D. congolensis* as the pathogen infecting the widest range of taxa, including many wildlife species (Ringwaldt et al. 2021).

### Habitat alteration and shifts

The degradation of wildlife habitat changes not only the availability of areas commonly used for nesting, foraging, and roosting. It can alter interspecies interactions and exacerbate other life history stressors (i.e., reproduction, migration). In the face of heat stress, animals have been shown to disperse in pursuit of more suitable microclimates (i.e., shaded areas, subterranean refugia) or alternative sources of water (Kamau and Maloiy 1985; Moore et al. 2018; van de Ven et al. 2019; Sauer et al. 2023). Increased exposure to pathogens sourced in livestock and other domestic animals has been documented in displaced wildlife. The SARS-CoV-2 pandemic has demonstrated the ease with which pathogens move between human populations and wildlife, particularly those that are synanthropic (e.g., white-tailed deer) (Kuchipudi et al. 2021; Cool et al. 2022).

Due to melting of sea ice, Arctic mammals are increasingly forced to spend summer months on land. This phenomenon has been associated with nutritional stress and increased exposure to both novel pathogens and heavy metal contaminants (Bourque et al. 2020; Fry et al. 2023; Neuman-Lee et al. 2017). These changes correspond to immune changes, including altered leukocyte distribution (i.e., elevated neutrophil: lymphocyte (N: L) ratio) (Whiteman et al. 2019; Bourque et al. 2020). This immunologic indicator is reported to shift in other species experiencing habitat shifts. For example, the N: L ratios of wild roe deer (Capreolus capreolus) were higher during years in which habitat quality was compromised (Carbillet et al. 2019). Similarly, WBC counts from multiple vampire bat populations revealed higher N: L ratios in those residing at the northern and southern edge of their geographic range, highlighting the influence of environmental surroundings and importance of accounting for within-species variability (Becker et al 2019).

Habitat alteration and shifts are particularly detrimental to populations already suffering from population fragmentation. Destruction of movement corridors reduces connectivity and can, at larger scales, disrupt migration patterns. Across scales, a decrease in genetic exchange can decrease immunogenetic diversity and compromise infection tolerance, particularly in the face of novel pathogenic threats driven by altered interspecies interactions. Using island amphibian populations to represent a naturally fragmented system, researchers described decreased MHC class II diversity and increased parasite richness compared to mainland populations (Belasen et al. 2019).

The impact of heat stress, drought, and altered weather patterns on primary patterns not only contributes to habitat degradation but also can disrupt trophic networks from the bottom up, exacerbating nutritional stress. Altered resource availability may result from a decrease in the quantity (i.e., drought-induced shortage) or quality (i.e., nutrient/quality decline) of a species’ diet. Studies examining the nutrient content of drought- and heat-stressed plants have shown decreased micronutrient levels and altered secondary metabolites resulting from disrupted nutrient uptake and dissemination (Choukri et al. 2020; Matías et al. 2021). Altered availability of native plants may push animals to consume non-native plants, which may be lower in nutritional quality and drive in energy imbalances that exacerbate immunosuppression (Pulscher et al. 2021).

Long-term field studies of Australian flying foxes (*Pteropus* spp.) were critical to demonstrating behavioral changes stemming from both climatic and land-use drivers that, in turn, predicted Hendra virus spillover events into horses (Eby et al. 2023). Increased viral spillover risks were in part due to the role these environmental changes played in causing food shortages for bat hosts and its association with increased viral shedding during energetically costly winter, likely driven by nutritional stress–induced immunosuppression (Becker, Eby, et al. 2023; Lagergren et al. 2023). Similarly, in the Nipah virus system, long-term field studies were central in establishing the role of winter temperature in being a primary predictor of spillovers from *Pteropus medius* bats into humans (McKee et al. 2021).

## Cascading risks: climate change as a threat multiplier

While the examples presented above illustrate the wide-ranging immunological impacts of climatic stress across biological scales, abiotic threats rarely occur in isolation. Wildlife are increasingly exposed to multiple simultaneous stressors, capable of compounding and amplifying immunological strain (Plowright et al. 2011; Becker et al. 2020). Predicting the ways in which abiotic stress may exacerbate susceptibility to other conditions (i.e., viral infection, habitat displacement) is complicated not only by knowledge gaps surrounding immune mechanisms, but also by differences in taxonomy, geography, and life history events. For example, long-term climatic shifts can trigger misalignment of seasonal environmental cues and critical life history events (e.g., migration, hibernation, reproduction)—evidence from long-term datasets (>20 years) demonstrates substantial phenological shifts across wildlife taxa (e.g., a 22-day reproductive delay in African wild dogs (*Lycaon pictus*), a 38-day delay in marmots' (*Marmota flaviventris*) emergence from hibernation, and a 16-day migration delay in Mexican free-tailed bats (*Tadarida brasiliensis*) (Inouye et al. 2000; Haest et al. 2021; Abrahms et al. 2022). Shifts in migration timing can shape community dynamics at stopover sites or breeding grounds, exposing animals to novel pathogens during periods of immunological vulnerability and energy deficit (Bauer et al. 2008; Hegemann et al. 2018; Haest et al. 2021). Perturbations to reproductive cycles can heighten the risk of infection for young animals, rendering them susceptible to enzootic pathogens in between maternal antibody decline and maturation of their own immune systems (Hasselquist and Nilsson 2008; Addison et al. 2009).

In the following section, we explore the complexity of characterizing the impacts of multiple interacting stressors, sometimes referred to as cascading risk pathways (Semenza et al. 2022). We use heavy metal contaminants as a case study to illustrate the ways that climate change–associated hazards interact with concurrent ecological pressures to exacerbate wildlife exposure to such contaminants. Heavy metals are naturally occurring in the environment, with some essential metals (i.e., zinc (Zn) and iron (Fe)) being required for biochemical and physiological functions. However, other non-essential metals (i.e., cadmium (Cd), mercury (Hg), arsenic (As), and lead (Pb)) have high degrees of toxicity and carcinogenicity, even at low levels of exposure, making them of high public and environmental health concern (Tchounwou et al. 2012). Floods and extreme weather events can promote mobilization of heavy metals (i.e., Pb, Hg, As, Cd) by causing excess runoff from industrial or agricultural sites (Soltaninia et al. 2024; Yang et al. 2024). Wildfires can also aerosolize or concentrate heavy metals sequestered in vegetation and topsoil in recently burned areas (Kumar et al. 2018).

The acute immunological consequences of these metals are likely underappreciated due to their sublethal and cumulative impacts and can have wide-reaching impacts across biological systems. Several studies demonstrate compromised reproductive indices in wild birds (e.g., hatchling survival) exposed to environmental metals (Ding et al. 2020; Sens et al. 2003). Frogs (*Bufo raddei*) alter reproductive investment in the face of chronic heavy metal exposure, possibly at the expense of population stability (Zhang et al. 2018). More recent attention has focused on the impact of these non-essential metals on immune regulation and control of infections. For example, Cd and Hg can cause immune suppression through dysregulation and increased apoptosis of immune cells, impact proliferation and differentiation of T and B cells, and alter cytokine expression (Rice et al. 2014; Wang et al. 2021), which may lead to increased susceptibility to or severity of infectious pathogens. Heavy metal exposure may also impact viral replication and shedding. An i*n vitro* study found pre-Cd treatment of canine-derived kidney cells increased influenza virus replication and cell oxidative stress in a dose-dependent manner (Checconi et al. 2013).

**Fig. 3:**
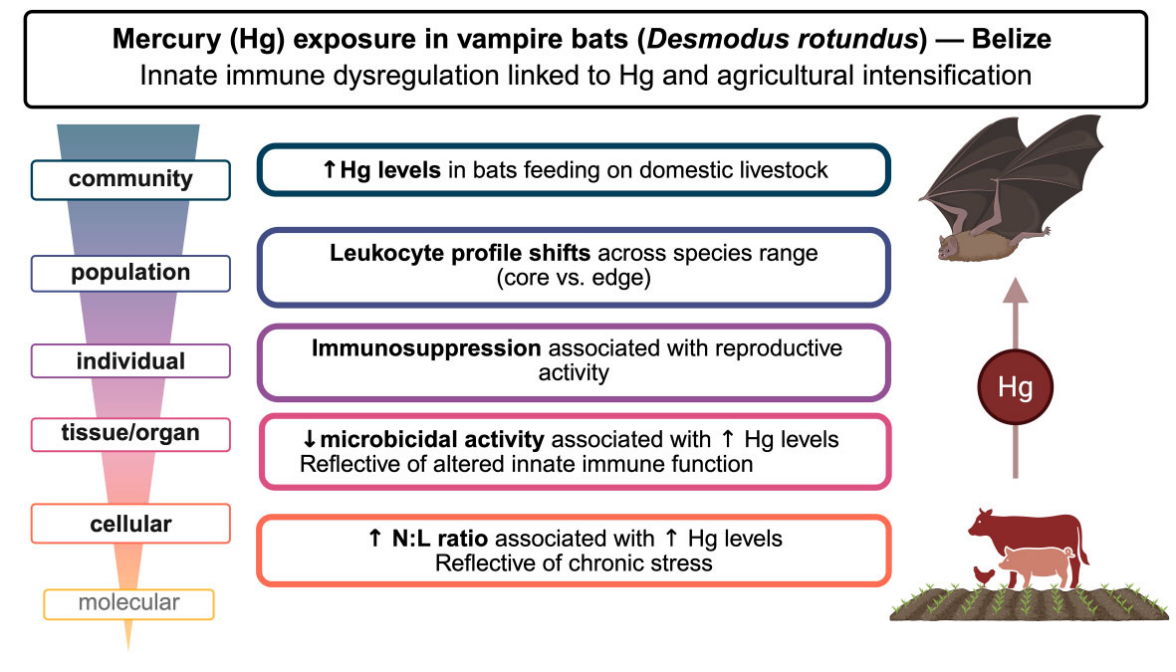
Immune dysfunction in vampire bats (*Desmodus rotundus*) exposed to mercury (Hg) and agricultural intensification in Belize. The accumulation of mercury (Hg) in common vampire bats foraging on livestock was associated with cellular signatures of chronic stress and impaired innate immune function. Agricultural intensification has increased the availability of livestock and resulted in conversion of forested areas to those used for grazing. Across the species’ geographic range, leukocyte profiles differed between bats at the core vs the edge of the range. Additional evidence of immunosuppression was identified in reproductively active animals, underscoring one of the mechanisms by which heavy metals can threaten long-term population stability. Created in BioRender.

In a wildlife context, studies of Hg exposure among free-ranging bats found higher fur Hg concentrations were associated with weaker innate immunity (Becker et al. 2017, 2021), which may make hosts more susceptible to infections (Fig. 3). Epidemiological studies of wild waterfowl found a positive relationship between avian influenza seroprevalence and Hg exposure (Teitelbaum et al. 2022). However, the impact of heavy metal exposure on pathogen susceptibility likely depends on pathogen type (i.e., bacteria, protozoa, virus) and host species (Becker et al. 2021). Nonetheless, heavy metal exposure can have significant direct and indirect effects on vertebrate immunity. When paired with other acute stressors, such as heat stress or nutritional stressors, heavy metal exposure may also result in significant population-level impacts through transgenerational epigenetic modification or reproductive losses (Zhang et al. 2018). Driven by the growing body of evidence describing heavy metal exposure in wildlife, bats and birds have been suggested as useful sentinel species for heavy metal contaminants throughout ecosystems (Gulson et al. 2009; Zukal et al. 2015; Pulscher et al. 2020).

## Expanding tools for comparative immunology in wildlife

### Leveraging historical samples and data

Collaborations among research groups enable the use of existing sampling efforts to maximize data obtained from free-ranging animals (Simonis et al. 2025). For instance, studies characterizing the virome and genetic diversity of museum specimens have revealed critical information about the evolutionary and ecological trajectories of many species and the pathogens they interact with over time (Colella et al. 2021). Similar efforts examining immunogenetic diversity across populations experiencing different pressures could provide insights into selective pressures or genetic bottlenecks. In attempts to characterize genomic erosion in the critically endangered orange-bellied parrot (*Neophema chrysogaster*) following multiple population crashes, researchers identified a temporal reduction in TLR diversity when comparing contemporary genomes (2014–2020) to those obtained from natural history collections (archived between 1843–1986) (Silver et al. 2025). These results highlight the importance of investing in the establishment and maintenance of biorepositories for future studies using banked specimens, now further aided by creation of “extended specimens” using advanced imaging and other approaches to facilitate digital access (Colella et al. 2023; Lendemer et al. 2020).

Future opportunities in harmonizing data across climate change–focused studies will require integration of transdisciplinary data. One such effort, ClimEx, provides a handbook guiding data collection, management, and reporting for experimental studies focused on climate change (largely focused on plants and herbivorous animals) (Halbritter et al. 2020). Adaptation of this system to reporting requirements for vertebrate responses to climate change (i.e., physiology, immunology, phenology, demography) would greatly benefit data re-use and synthesis efforts. Coupling such a framework with harmonized immunological data would facilitate assessments of immunogenetic diversity indices (e.g., an immunogenetic diversity database featuring MHC sequences, as proposed by (Cortazar‐Chinarro et al. 2025)).

### Rising -omics availability

Determining changes in gene expression via transcriptomic analysis can provide granular insights into host responses to infection, stress, and other challenges—both exogenous and endogenous (Arnold et al. 2018; Dou et al. 2021; Shi et al. 2025). These analyses necessitate mapping orthologous genes to a reference genome—ideally the annotated genome of the target species—but the rapid increase in available genomes makes identification of gene orthologs from closely related species a viable alternative approach (Zhu et al. 2014; Peel et al. 2022; Ponomarenko et al. 2022). RNA sequencing is increasingly used in wildlife studies characterizing transcriptional differences in response to abiotic stress (e.g., heat-stressed turtles demonstrate elevated expression of heat shock proteins (HSP70 and HSP90) (Tedeschi et al. 2015; Sparks et al. 2022)). Proteomic profiling has been applied to questions investigating immunity and infection in wildlife, with proteomic signatures of infection and immunotolerance identified in free-ranging bats (Neely et al. 2021; Becker et al. 2022). The rise of epigenetics also provides opportunities to evaluate epigenetic modifications across diverse taxa—unique methylation signatures have been identified in association with food availability in baboons (*Papio cynocephalus*) (Lea et al. 2016), anthropogenic stress in killer whales (*Orcinus orca*) (Crossman et al. 2021), parasite infection in house sparrows (*Passer domesticus*) (Lundregan et al. 2022), dehydration and anoxia in wood frogs (*Rana sylvatica*) (Kupakuwana et al. 2024), and heavy metal exposure in great tits (*Parus major*) (Mäkinen et al. 2019).

### Immunological cell profiling

A more resource-intensive approach, flow cytometry, can provide granular insights into multiple markers of cellular immunity status, including assays that characterize cell vitality (to detect necrosis and apoptosis), immunophenotypes, surface markers, and cell proliferation (see overview by Song & Lee 2024). It can also be used for fluorescence-based cell sorting, with some success reported applying commercially available antibodies to use in wildlife species (e.g., characterizing T cell populations in African lions (*Panthera leo*) (Basu et al. 2010; Sylvester et al. 2018). In free-ranging wildlife, flow cytometry has had limited applications investigating the impacts of abiotic stress. It has, however, been used to characterize immune profiles in trypanosome-infected woylies (*Bettongia penicillata*) as well as in green sea turtles (*Chelonia mydas*) facing multiple stressors (i.e., habitat degradation and papillomavirus infection) (Hing et al. 2016; Sposato et al. 2021). It has also been used for serodiagnostic assay development to characterize exposure of wildlife to plague (*Yersinia pestis*) (Chandler et al. 2018) and to characterize the cellular immune landscape of multiple wildlife species in captive settings (e.g., Egyptian fruit bats (*Rousettus aegyptiacus*)) (Friedrichs et al. 2022)). Approaches for overcoming species-specific limitations in available reagents for flow cytometric assays are increasingly common (reviewed in Hunka et al. 2020).

### *In vitro* and *ex vivo* approaches

As a proxy for animal models, a growing suite of *in vitro* and *ex vivo* approaches facilitates the study of immune function in diverse taxa with tractable platforms examining molecular mechanisms. Opportunistic samples collected from moribund or euthanized wildlife have been used to generate primary cell lines for use in immunology research (Banerjee et al. 2016). These may be derived from postmortem samples (e.g., lung, kidney), but non-lethal methods are also described, such as wing punches from field-caught bats (Alcock et al. 2024). The development of cell lines is often guided by experimental objectives—such as viral susceptibility testing or comparative transcriptomic profiling. Some cell types (e.g., fibroblasts) may be more amenable to isolation and serial passage than others, and cell-specific growth requirements may limit the types of stressors that can be experimentally applied. These tools are increasingly common in wildlife conservation, including efforts to generate pluripotent stem cell-derived organoids from endangered species (Zywitza et al. 2022; Johnson 2024). Cell viability is often a limiting factor in selecting stressor/cell line combinations, with cell lines derived from some taxa (e.g., bats) demonstrating greater thermal tolerance than those from others (e.g., birds, mice) (Chionh et al. 2019). These techniques afford immunologists an important bridge for translating findings across species, particularly those lacking appropriate diagnostic reagents, clinical data, or tractable *in vivo* models.

### Non-traditional animal models *(in vivo* methods)

Captive breeding colonies of wild-sourced animals are increasingly common, particularly for studies interrogating functional immune outcomes in the face of simulated stress and/or infection (i.e., nutritional stress and viral infection in captive Jamaican fruit bats (*Artibeus jamaicensis*)) (Crowley et al. 2024; Falvo et al. 2025). A captive Egyptian fruit bat (*Rousettus aegyptiacus*) colony has revealed invaluable insights surrounding the maintenance and transmission of Marburg virus, along with the ways that experimental stress and coinfection modulate susceptibility to infection (Amman et al. 2020; Guito et al. 2024; Schuh et al. 2025). Wild-caught animals transported into a laboratory setting are also used for studies examining susceptibility to abiotic stress and/or pathogen infection. Wild-caught four-striped field mice (*Rhabdomys dilectus*) and Namaqua rock mice (*Micaelamys namaquensis*) subjected to experimental heat stress showed signs of oxidative damage (malondialdehyde and protein carbonyl) as well as heightened antioxidant defenses (superoxide dismutase and total antioxidant capacity) across tissue homogenates representing multiple organ systems (liver, kidney, and brain) (Jacobs et al. 2020, 2021).

Beyond mammalian models, the *in vivo* study of *Mycoplasma gallisepticum* in house finches (*Haemorhous mexicanus*) has evolved into a valuable model system for investigating drivers of susceptibility (including co-infection), virulence evolution, and transmission (Love et al. 2016; Fleming-Davies et al. 2018; Bale et al. 2020). Controlled administration of abiotic stress in established outbred animal models enables mechanistic insights into how multiple stressors, including pathogen infection, influence immune outcomes across time (ranging from acute to chronic stress) and immune components (e.g., innate immunity, humoral responses) (Inglis 2007; Vonaesch et al. 2018; Barrero Guevara et al. 2025; Rozen‐Rechels et al. 2020; Udino et al. 2024). Translating results from model organisms, including domestic species, can aid in testing hypotheses about stressors’ interactions and chronicity while also guiding predictions relevant to wildlife immunity.

## A roadmap of current and future focus areas for studying wildlife immune responses to climate change: livestock as a model

Despite growing recognition that climate change alters wildlife health and disease dynamics, the role of the immune system is often overlooked in vulnerability assessments. We argue that immune function is more than a collection of physiological indicators—it represents a critical lens for identifying when, where, and in which taxa climate-related health risks may be greatest. Our ability to maximize immunological assessment of climate change impacts across taxa requires an integrative framework spanning biological scales—from molecular to community levels. Such a framework would include defining baseline immune function in each population, identifying the most relevant biomarkers of stress and immune dysregulation, and optimizing minimally invasive sampling strategies to maximize early detection. It will also require integrating experimental and observational study results, leveraging emerging tools and methodologies, and integrating findings across disciplines to better characterize mechanistic underpinnings of immunosuppression and disease susceptibility. Here, we highlight livestock as a tractable and flexible, yet underused, model for identifying diagnostic biomarkers relevant to wildlife climate vulnerability.

### Livestock systems span taxa and ecological contexts

A considerable body of work exists examining immunological impacts of abiotic stress in livestock, involving a broad range of taxa—from fish (e.g., aquaculture) to mammals (e.g., cows and pigs) and avian species (e.g., poultry). The wide range of husbandry and management practices used in these systems represent varying levels of climate control (i.e., free-range versus indoors), providing high-resolution environmental data over extended periods of time without sacrificing ecological relevance. Further, habituation to human presence minimizes confounding stressors during sample collection. Optimization of diagnostic schemes and mechanistic understanding of pathogenesis can be aided by pairing these natural systems with experimental work in relevant established animal models (i.e., sheep and swine bred for orthopedic and cardiovascular research, respectively).

### Diagnostic tools and identification of biomarkers

Immunological markers identified in domestic species offer promising and scalable early-warning indicators of vulnerability that could be used to complement other physiological or demographic metrics in wildlife health monitoring assessments, but these require additional validation across species and sample types. Identification of biomarkers via multiple -omics platforms permit optimization of antemortem sampling regimes to minimize direct handling. Several non-coding RNAs, including microRNA (miRNA) levels, are responsive to infection, inflammation, and physiological stress and can often be detected earlier in the course of illness than historically used protein biomarkers. Changes in miRNA expression have been described in heat-stressed animals representing several livestock species including (but not limited to): cattle, sheep, goats, pigs, fish, and chickens (Hao et al. 2016; Huang et al. 2018; Li et al. 2018; Fan et al. 2021; Goel et al. 2021; Yadav et al. 2021; Liu et al. 2024). Studies examining genotypic-phenotypic correlates in cattle, pigs, chickens, and fish have identified candidate biomarkers corresponding with immunosuppression, inflammation, and other correlates of compromised health (i.e., decreased food intake, activity—and, in dairy cattle, decreased milk output (Cowley et al. 2015; Tian et al. 2016)). Similarly, extensive work in the aquaculture industry could shed light into immunological mechanisms of pathogen sensitivity driven by abiotic stressors unique to aquatic environments (e.g., pH, dissolved oxygen) (**Abdel-Tawwab et al*. 2015 ; *Singh et al*. 2016*; Zhou et al. 2021).

### Translational case studies for use in wildlife research

Studies in cattle have demonstrated increased susceptibility to infection associated with abiotic stress. For example, heat stress is a driver of “leaky gut,” condition marked by gastroenteritis and increased movement of bacteria, endotoxins, and digestive enzymes across the small intestinal epithelial barrier (Koch et al 2019). Similarly, “shipping fever”—a term used to describe the constellation of pathogens associated with pneumonia following transport-associated immunosuppression in beef cattle—could be used as a model to explore the mechanisms driving increased bacterial pneumonia in heat-stressed wild ungulates (e.g., Saiga antelope, bighorn sheep). Examples from the livestock literature also reveal insights into heat stress impacts on adaptive immune responses. For example, studies in broiler chickens and neonatal dairy cows demonstrated that heat-stressed animals had greater dysregulation in lymphocyte ratios and less robust neutralizing antibody responses following vaccination than those held in thermoneutral environments (Honda et al. 2015; Marrero et al. 2021). Nutritional stress is another active area of investigation in the agricultural sector, with quantification of feed intake correlated to changes in environmental conditions and/or life history stages.

Studies in livestock already offer insights into how abiotic stress impacts immunity across generations and developmental stages. Thermal stress during pregnancy and lactation disrupts the physiology and immunity of both dam and offspring, possibly impacting population age structure or threatening climate change resilience across life stages. Research on neonatal dairy cows indicates that those born to heat-stressed dams suffer from delays in immune system development (Marrero et al. 2021; Fogel et al. 2023). Similarly, much of the work exploring maternal antibody decay has been published by scientists in the agricultural sector (e.g., vulnerability to infection in recently weaned dairy calves). These data could inform investigations into maternal antibody decline and shifts in pathogen susceptibility in young wildlife. Leveraging existing research taking place in the livestock sector represents a unique opportunity to work across disciplines to enhance our understanding of immunology across both domestic and free-ranging animals experiencing shared ecological stressors associated with climate change.

## Conclusion

Climate change impacts wildlife immunity across biological scales—from perturbations in cytokine signaling at the cellular level to altered infection dynamics at the population and community levels. These effects are widespread and often difficult to quantify due to the complexity of disrupted biotic and abiotic interactions. To better understand and monitor immune responses to abiotic stressors in wildlife, we must adopt a framework that leverages mechanistic advances made in other species to guide diagnostic optimization and risk assessment. The agricultural sector has invested significant resources in identifying and mitigating climate-related morbidity and mortality across a broad range of taxa, representing a unique opportunity to draw parallels and translate approaches across disciplines. Sustained progress in climate-health research will require stable support structures—both public and private—that prioritize interdisciplinary collaboration and long-term capacity building. Meeting these challenges will require research frameworks that are as adaptive as the species we study, providing actionable guidance for both wildlife conservation and public health.

## Data Availability

N/A. There is no supplementary data associated with this work.

## Notes

From the symposium “Applied ecoimmunology in wildlife health and conservation” presented at the annual meeting of the Society for Integrative and Comparative Biology, January 3–7th , 2025.
